# Role of some members of chemokine/cytokine network in the pathogenesis of thalassemia and sickle cell hemoglobinopathies: a mini review

**DOI:** 10.1186/s40164-019-0145-x

**Published:** 2019-09-11

**Authors:** Zahra Mousavi, Zinat Yazdani, Alireza Moradabadi, Fatemeh Hoseinpourkasgari, Gholamhossein Hassanshahi

**Affiliations:** 1 Department of Hematology and Medical Laboratory Sciences, Iranshahr University of Medical Sciences, Iranshahr, Iran; 2 Department of Hematology and Blood Banking, Kerman University of Medical Sciences, Kerman, Iran; 3 Department of Hematology, School of Paramedicine, Arak University of Medical Science, Arak, Iran; 4 Molecular Medicine Research Center, Rafsanjan University of Medical Sciences, Rafsanjan, Iran; 5 Department of Immunology, Rafsanjan University of Medical Sciences, Rafsanjan, Iran

**Keywords:** Hemoglobinopathy, Sickle-cell disease (SCD), Cytokine, Chemokine

## Abstract

The word of hemoglobinopathy is described for an array of disorders that affecting hemoglobin (Hb) functions. Hb is a molecule with 68 kDa molecular weight, serving as oxygen carrying metalloprotein. Hemoglobinopathy includes a wide range of Hb structural deficits varying from thalassemia to sickle cell disease. Cyto-chemokine network members are pivotally involved in the pathogenesis of hemoglobinopathies, however, the exact role of these mediators in the development of these disorders yet to be well addressed. Cytokines and chemokines are generated by inflamed endothelial cells that promote the expression of their respected receptors and further activate NF-κβ, recruit red blood cells (RBCs) and white blood cells (WBCs) toward the inflamed endothelium. Therefore, due to critical roles played by the cyto-chemokine network in several aspects of hemoglobinopathies pathophysiology including apoptosis of endothelial cells, RBC, WBC and etc.…, in the present review, we focused on the critical parts played by this network in the pathogenesis of hemoglobinopathies.

## Introduction

The word of hemoglobinopathy defines a wide spectrum of disorders which affecting hemoglobin (Hb) activities [1]. Hemoglobin is a molecule with molecular weight of 68 kDa that serves as an oxygen transporter metalloprotein and is located in red blood cells (RBC). The normal type of Hb bio-structure includes two alpha and two beta globin chains that are linked covalently to four heme molecules as oxygen building prosthetic groups [2]. The HbA form of Hb has with (α_2_β_2_) formula and constitutes approximately 95% of total Hb in healthy adults. Other minor Hbs are HbA2 (α_2_δ_2_) (< 3.5%) and HbF (α_2_γ_2_) (< 2%). The early embryonic Hbs are also Portland I (ζ_2_γ_2_), Hb Gower I (ζ_2_ε_2_) as well as Hb Gower II (α_2_ε_2_) [3]. The HbF form or fetal Hb is generated and developed later and is the principle oxygen carrying moiety during the fetalic period. A genetic switch triggers the synthesis of the β-globin chain which led to elevated HbA along with the HbF reduction further birth [4]. A broad spectrum of Hb disorders is described and classified into three various categories of hemoglobinopathies, thalassemia and delta/beta thalassemia. Therefore, most of the studies have described thalassemia as a subtype of hemoglobinopathy [5]. Hemoglobinopathies are most often due to structural variations in either alpha or beta globin chain, thus, they may generally arise either by single nucleotide polymorphisms (SNP) or point mutations. Hemoglobinopathies are mainly differed from thalassemia by the fact that in thalassemia the production of whole alpha or beta globin chains are disrupted. Furthermore, the delta/beta thalassemia and hereditary persistence of fetal Hb (HPFH) are both clinically identified by the elevated levels of HbF, even in adulthood. The locus of the alpha globin gene is on the chromosome 16, whereas gene for the beta globin is located on the chromosome number 11 [2]. It has been well evidenced that approximately 7% of the world population are carriers of Hb disorders and both of α- and β forms are the main types of thalassemia. In Africa, Arab nations, and, more frequently, South-East Asia, Mediterranean countries, South-East Europe, and Asia thalassemia are prevalent. The main structural hemoglobin variants are HbS, HbE and HbC, worldwide. The predominant clinical manifestations for hemoglobinopathies are varied from mild hypochromic anemia, moderate hematological disorders to severe, life-long, and transfusion dependent anemia with multi-organ involvement. If available, the stem-cell transplantation is of the most preferred, treatments proposed for the severe types of thalassemia. Periodical blood transfusion (e.g. washed packed red blood cell) for life in combination with iron chelation are also defined as supportative therapy. The sickle-cell disease (SCD) as the most frequent type of hemoglobinopathy is treated with analgesics, antibiotics, angiotensin converting enzyme (ACE) inhibitors and hydroxyurea [1]. The SCD is now recognized as a complex disease characterized by acute and chronic inflammation [6]. In SCD, further deoxygenation, HbS forms rigid, intracellular insoluble polymers, in which the affected RBCs form characteristics of sickle shape, and in turn lead to vaso-occlusive phenomena [7, 8]. The featured complication of SCD is vaso-occlusion (VOC) and further to these event blood vessels are blocked by injured erythrocytes. Further, both chronic and acute clinical states of vaso-occlusion, ischemia and hypoxia are of potentially remarkable multiple sequels are evident. Several other complications, including stroke, splenic infarction, infection, priapism, acute chest syndrome, kidney failure, pulmonary hypertension, necrosis of bone, pain crisis and retinopathy are also prevalent in SCD [9–11]. The microcirculation occlusions (both clinical and subclinical), hemolysis and infections are of crucial events (inflammatory and non-inflammatory) that are involved in promoting of the cytokines and acute-phase proteins production and further secretion. Predominantly paramount parts played by cytokines in SCD are subject of multiple research programs [12]. The events of cytokines release following infections, endothelial cell activation, and other injurious and clinical states play pivotal roles in the pathophysiology of microvascular occlusion in SCD [13]. The mechanisms by which vaso-occlusion crisis commenced are complex in nature as well as multifactorial, nonetheless, evidences proposed that adhesion of sickled RBC is drastically important in the overall VOC processes [9]. A huge body of evidence indicated that homozygotic SS form of RBC population, but not normal RBC population, are able to bind to several targets such as vascular endothelial cells (EC), platelets, the vascular adhesive proteins thrombospondin (TSP) and laminin within the extra-cellular matrix. The adhesion of homozygotic SS RBCs to these cellular or molecular targets is almost believed that at least in part initiates the phenomenon of vaso-occlusion [14]. The endothelium is also adversely affected by SCD and may contribute to vaso-occlusion. The continuous injuries raised by pro-inflammatory cytokines and iron overload are assumed to activate the vascular endothelium, in turn, compromise vascular integrity as well as stimulate both RBC and leukocytes adhesion within the vascular beds [15]. Vast level of hemolysis is also associated by the release of hemoglobin from intra cellular sources. Erythrocytes also claimed to serve as nitric oxide (NO) scavenger, and hence altering vascular tone and dilation that is followed by disrupted blood flow [16]. Platelets alongside with the coagulation cascade factors are then activated in SCD and they could also affect the endothelium and contribute to the phenomena of vaso-occlusion and hypoxia in these circumstances [17].

The leading mechanism for abnormal adhesiveness involves characteristics of the RBCs membranes such as (integrin α_4_β_1_, glycoprotein CD36), endothelial membrane components [V-CAM-1 (vascular-cell adhesion molecule-1), CD36, fibronectin] and plasma factors, including cytokines [18].

### The interaction between WBCs, RBCs, and endothelium

Incubation of the homozygotic SS from RBCs with endothelial cells in several different in vitro models reported to elevate the expression of VCAM-1, ICAM-1 and E-selectin, which are the EC receptors of the blood cell adhesion molecules very late antigen-4 (VLA-4), b2-integrin and L-selectin, respectively, in parallel with IL-1 and TNF-α [6, 19]. Activation of monocytes in patients suffering from SCD could occur due to binding of either platelet or RBC to monocytes. Further activation, platelets are able to bind to and activate monocytes. The aggregated platelets and monocytes have been documented to play crucial parts role in vascular diseases [20, 21]. Experimental evidence proposes that the homozygotic SS form RBCs bind to vascular ECs by integrin complex α_4_β_1_. The α_4_β_1_ integrin receptors or VLA-4 could be activated by CXCL8 on SCD reticulocytes [13], which is secreted by both Endothelial Cells (ECs) and WBCs in injured regions of inflammed endothelium [22]. In SCD, CXCL8 elevates the adherence of homozygotic SS form RBCs to the endothelium via activated α_4_β_1_ on SCD reticulocyte and endothelial surface-associated fibronectin. The enhanced level of CXCL8 in SCD patients serves as a migratory factor for neutrophils as well as contributes to the initiation of vaso-occlusion and pain crisis of SCD [13, 23]. It has also been observed that activated platelets of patients with SCD produce thrombospondin and members of cytokine/chemokine network particularly IL-1 and CXCL8 that contribute to the starting of vascular occlusions and IL-1 activates the vascular endothelium as well [24–26]. RBCs and ECs of SCD patients express glycoprotein IV (CD36) on their membrane, which serves as a specific receptor for TSP. Plasma TSP which is released from activated platelets attaches to the quiescent and activated ECs that is therefore believed to promote adhesion of SS-RBCs to the endothelium in vivo. Additionally, TSP attaches to endothelial integrin complex αvβ3 to sulfated glycans on the sickled cells surfaces [27, 28]. ICAM-1, VCAM-1, P-selectin and E-selectin and the procoagulant molecule tissue factor are all expressed on activated endothelium and play pivotal parts in the WBCs oriented locomotion and the promotion of thrombosis within the inflamed vascular system. In addition to tissue factor, VWF, phosphatidylserine (PS) exposure on activated monocytes and endothelium in SCD might, therefore, be considered as the determinants for VOC crisis [29, 30]. Evidence are in favor of the concept that polymorphonuclear cells (PMNs) are critically involved in the pathophysiology of SCD. Firstly, an elevated number of PMNs is of the characteristics of SCD patients, and PMNs are related to the increased risk of early death, acute chest syndromes (ACSs), in addition to stroke [31]. Secondly, hydroxyurea (HU) abolishes the PMNs numbers and thus this parameter can explain a link between the beneficial effect of HU and SCD [32].

**Fig. 1:**
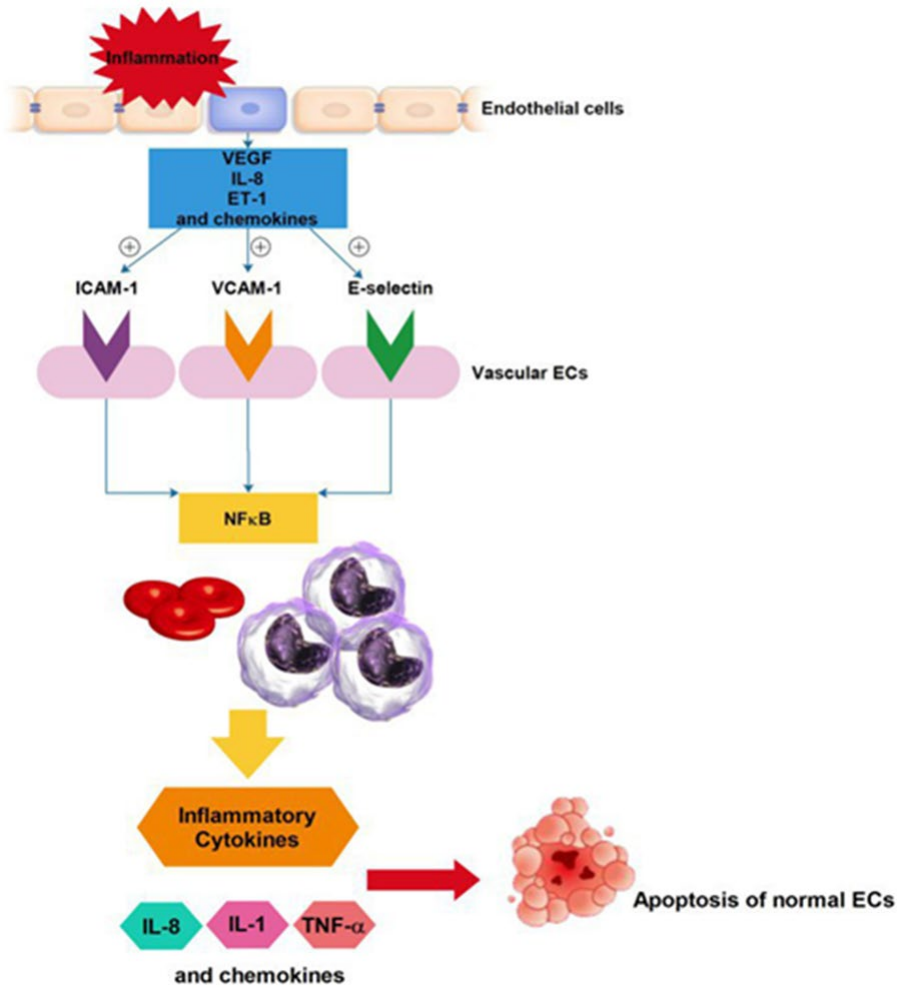
Demonstrates the potential interactions between homozygotic SS form RBCs of SCD and the blood vessel wall endothelium. *VEGF* vascular endothelial growth factor, *ET-1* endothelin-1, *V-CAM-1* vascular-cell adhesion molecule-1, *ICAM-1* intercellular adhesion molecule 1, *NF-κβ* nuclear factor-κβ, *IL* interleukin, *TNF-α* tumor necrosis factor-α

The novel finding of the intracellular mechanisms for ECs activation has addressed critical points concerning the establishment of therapy protocols. The anti-inflammatory impacts of glucocorticoids, sulfasalazine and aspirin are achieved by inhibition of NF-κβ signaling pathway, that is now defined as the most important stimulator for transcription of genes involved in ECs activation in combination whit multiple cytokines and adhesion molecules [33, 34]. HU attenuates RBC adhesiveness to endothelium, in addition to CD36 and integrin α_4_β_1_ expression on the RBC membrane. HU also attenuates the complication of the event of painful vaso-occlusive crisis not only by elevating the level of fetal hemoglobin but also via myelosuppression and further causing neutropenia [35, 36] (Fig. 1).

### Apoptosis of vascular endothelial cells

The phenomenon of apoptosis is described as the genetic based programmed cell death which participates in the pathogenesis of a broad range of disorders, varied from cancer, degenerative diseases of the nervous system autoimmune diseases, to cardiovascular diseases, and vascular disorders [37]. Cytokines play fundamental parts in dys-regulation of the apoptosis of endothelial. The endothelin-1 (ET-1) and VEGF are produced and secreted from a wide spectrum of inflamed cells during vascular insult or hypoxia and are defined as multifunctional growth factors [38, 39]. The elevated levels of VEGF promote the expression of ICAM-1, VCAM-1, and E-selectin on vascular ECs and activate NF-κβ pathway, which is a well-defined transcription factor that mediates vascular inflammation [40]. Therefore, the increased measures of VEGF during either steady state or SCD crisis could be assumed to recruit adhesive RBCs and WBCs derived from SCD patients to inflamed endothelium, in vivo [41]. Inflammatory cytokines including IL-1 and TNF-α are able to trigger apoptosis of normal ECs in vitro [42]. Levels of ET-1, VEGF, IL-1, and TNF-α are reported to be thus elevated in SCD patients which probably contribute to the vascular pathology of the disorder either positively via IL-1 and TNF-α or negatively by ET-1 and VEGF. These mediators play paramount parts in the normal apoptosis processes in endothelial and vascular smooth muscle cells [43].

There exist only a few articles which addressed the endothelial apoptosis in SCD, however, Solovery and colleagues reported enhanced percentage of activated circulating dead ECs (34%) with microvascular origin in SCD patients. On the basis of the previously mentioned content, these dead ECs could have been died from apoptosis, due to the induction of the endothelial apoptosis mediated by cytokines such as IL-1 and TNF-α. However, in ongoing investigations in SCD patients, only 30% of the circulating ECs have undergone apoptosis, while this percentage is approximately 60% in healthy controls. It appears that in SCD, the vascular endothelium is preserved from apoptosis, may be in response to the increased measures of anti-apoptotic agents, namely VEGF or ET-1. Solovey and co-workers proposed that induced VEGF plasma content participates in the induction of endothelial anti-apoptotic tone probably by stimulation of neovascularizing retinopathy in SCD patients [44–46]. Experimental evidences have revealed that SCD patients even in the steady state showed elevated cytokine production by activated endothelial cells [15, 43]. Increased adhesiveness of sickle to the cultured ECs has been observed even whereby cells were obtained from SCD patients during the steady state [47]. These cytokines were secreted by (i) activated endothelium (e.g. IL-1, IL-6, IL-8, TNF-α), (ii) activated platelets (e.g. IL-1, TNF-α), and (iii) monocytes and macrophages population (e.g. IL-1, TNF-α) further activation of the vascular endothelium [48]. These WBCs-derived cytokines also activate the RBC adhesiveness to the endothelium and further form a vicious cycle, in order to lead to the aggregation of more dense, irreversibly sickled RBCs, platelets and neutrophils, and finally VOC. Balancing between the steady state and crisis status is fragile and factors, such as infection or inflammation that cause further EC activation or increase in the RBCs adhesiveness may incorporate to the VOC event [49].

### Placental growth factor

A member of the VEGF superfamily which is potentially related to the SCD is placental growth factor (PlGF). PIGF initially is believed to be only produced and released from the placenta during pregnancy, however, it is also produced by erythropoietic cells in both the bone marrow and the peripheral blood [50]. PlGF is perhaps increased in SCD patients during steady-state and is even more elevated further vaso-occlusive crisis [51]. Although the reported increased levels of PIGF in SCD are lower than the measures in pregnancy, it still could have remarkably deep effects on the vasculature in SCD. In a similar fashion as VEGF, PlGF is also able to potently serve as an angiogenic factor and binds to VEGF receptors. It also appropriately activates monocytes of SCD patients, however, it appears to exert its own impacts that are pathologically relevant to SCD [52]. The activation of monocytes could further elevate monocyte adhesion to the endothelium as well as promotes their inflammatory factors production such as TNF-α and a variety of interleukins that are able to exacerbate endothelial cell dysfunction in parallel with leukocyte function. The effect of PlGF on retinopathy or sickle RBCs adhesion to the endothelium deserves to be fully elucidated [6].

## Conclusions

SCD is defined as a host of vascular perturbation with the involvement of endothelial cell dysfunction, leukocytosis, leukocyte activation and platelet activation.

It appears that cytokine-chemokine network is crucially involved in the pathogenesis of vaso-occlusive events which are described as important clinical symptoms of SCD by several possible mechanisms as following: (a) activates endothelium, (b)facilitates adhesion of RBCs and neutrophils to the endothelium, (c) causes attachment of neutrophils to the plasma fibronectin, (d) involves in progression of vascular intimal hyperplasia, (e) activates platelets, (f) induces the endothelin production, and (g) finally dysregulation of apoptosis. Studies have evidenced altered quantities of cytokines, chemokines and acute-phase proteins in steady-state of SCD patients.

Further research programs regarding the roles played by cytokines and chemokines in SCD may elucidate the pathogenesis of the disease and its complications and possibly aid to evaluation of the disease severity and prognosis. Immediate and rapid examination of inflammatory cytokines and chemokines through the steady state may probably serve as a beneficial test for the physician to intervene therapeutically with either hydroxyurea or erythropoietin in these patients.

## Data Availability

Please contact author for data requests.
